# Supplementary material for: Prevalent chromosome fusion in Vibrio cholerae O1
Source: Nat Commun. 2025 Jul 1;16:5830. doi: 10.1038/s41467-025-60699-0 (PMC12219848; doi:10.1038/s41467-025-60699-0)
Supplement: Supplementary file 1 — Supplementary Information [file 41467_2025_60699_MOESM1_ESM.pdf]

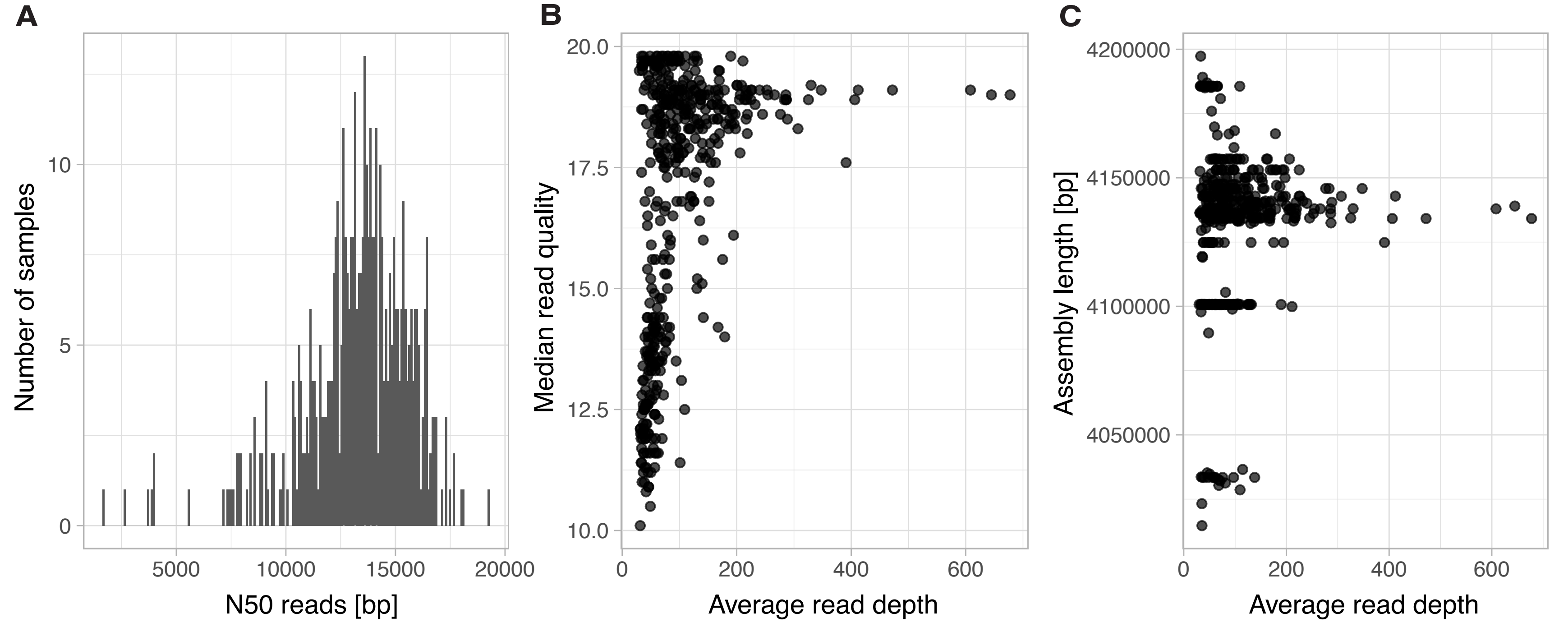

Figure S1: Quality measures of the genomes sequenced for this study. **A:** N50 of the reads per sample; **B:** Median read quality and average read depth; **C:** Assembly length and average read depth. Source data are provided as a Source Data.

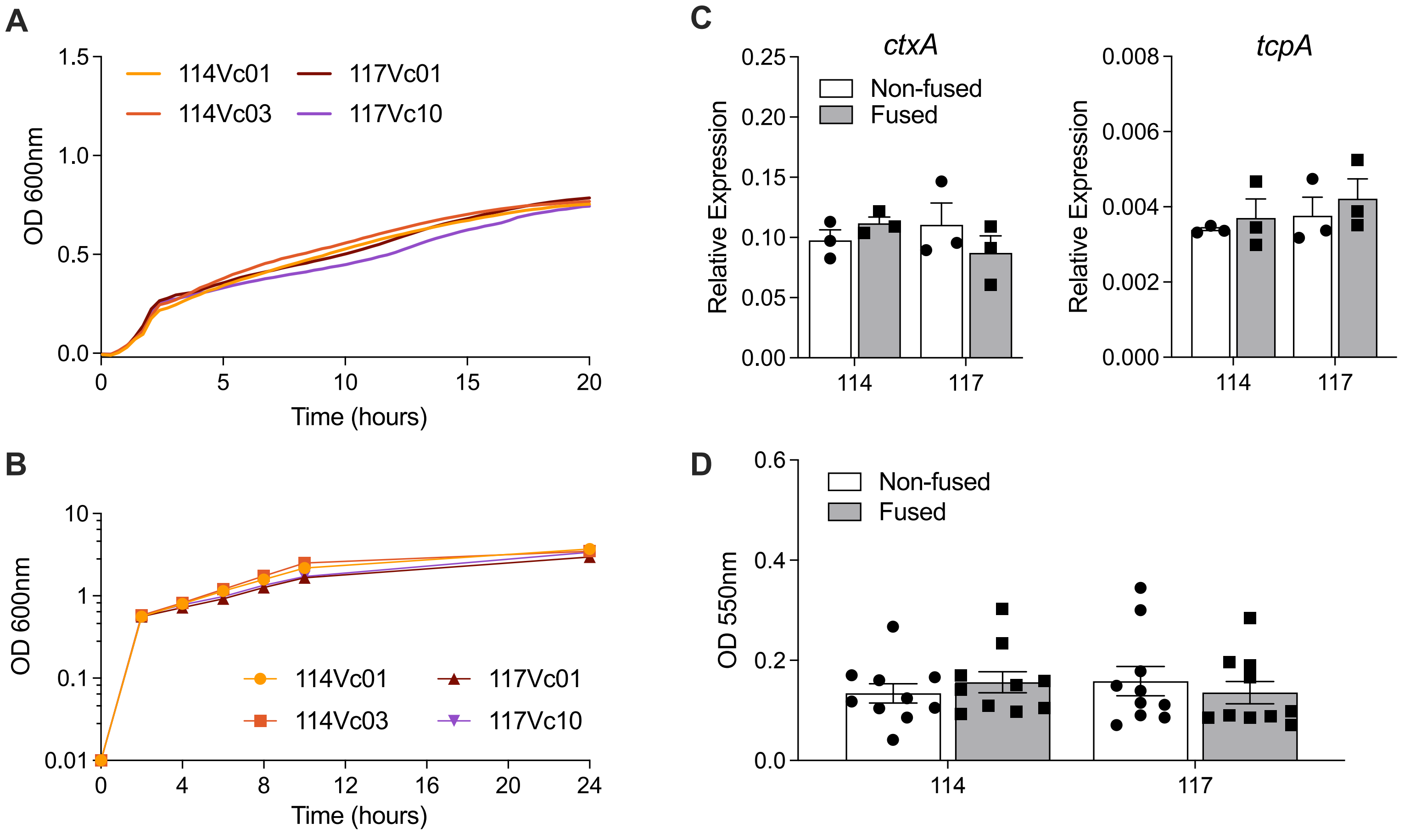

Figure S2: No evidence for phenotypic effect of fusion detected. Two pairs of strains were compared which were isolated from the same patient, each including one strain with a fused (114Vc03 and 117Vc01) and one with a non-fused (114Vc01 and 117Vc10) chromosome **A:** Growth curved measured in 96-well plates; **B:** Growth curves measured in a roller drum; **C:** Expression of the cholera toxin subunit A (ctxA) and the toxin co-regulated pilus sub-unit A (tcpA) measured by qPCR and **D:** Biofilm formation measured by Crystal violet staining. OD: Optical Density. Error bars indicate the standard error. Source data are provided as a Source Data file.

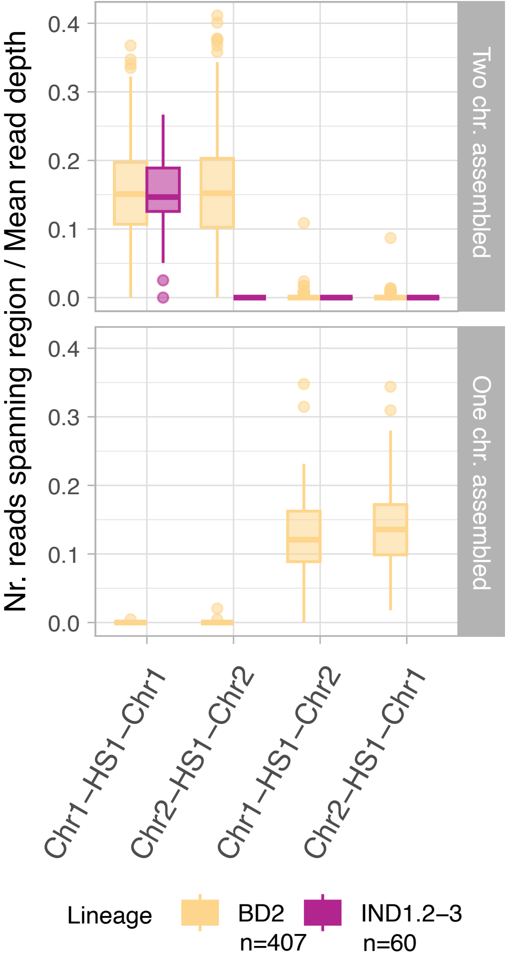

Figure S3: Number of reads spanning HS1 and 500bp flanking sequences on either chromosome (Chr1 or Chr2). The chromosomal structure inferred using the assembly is highly concordant with the reads, with reads spanning 'Chr1-HS1-Chr2' or 'Chr2-HS1-Chr1' being found almost exclusively in one-chromosome (fused) assemblies. An exception was the isolate 112Vc05, which assembled into two chromosomes, but for which we identified reads anchored on both chromosomes as well as one read anchored in chromosome 1 on both sides (i.e. a false-negative fusion in the assembly). All other nine isolates from the same patient assembled into one fused chromosome. The outlier, 112Vc05, likely also contains a fused chromosome, although we cannot exclude a mixed population of fused and non-fused genomes in this colony. Boxplots indicate the median and interquartile ranges with the whiskers representing the lower and upper interquartile range*1.5. Source data are provided as a Source Data file.

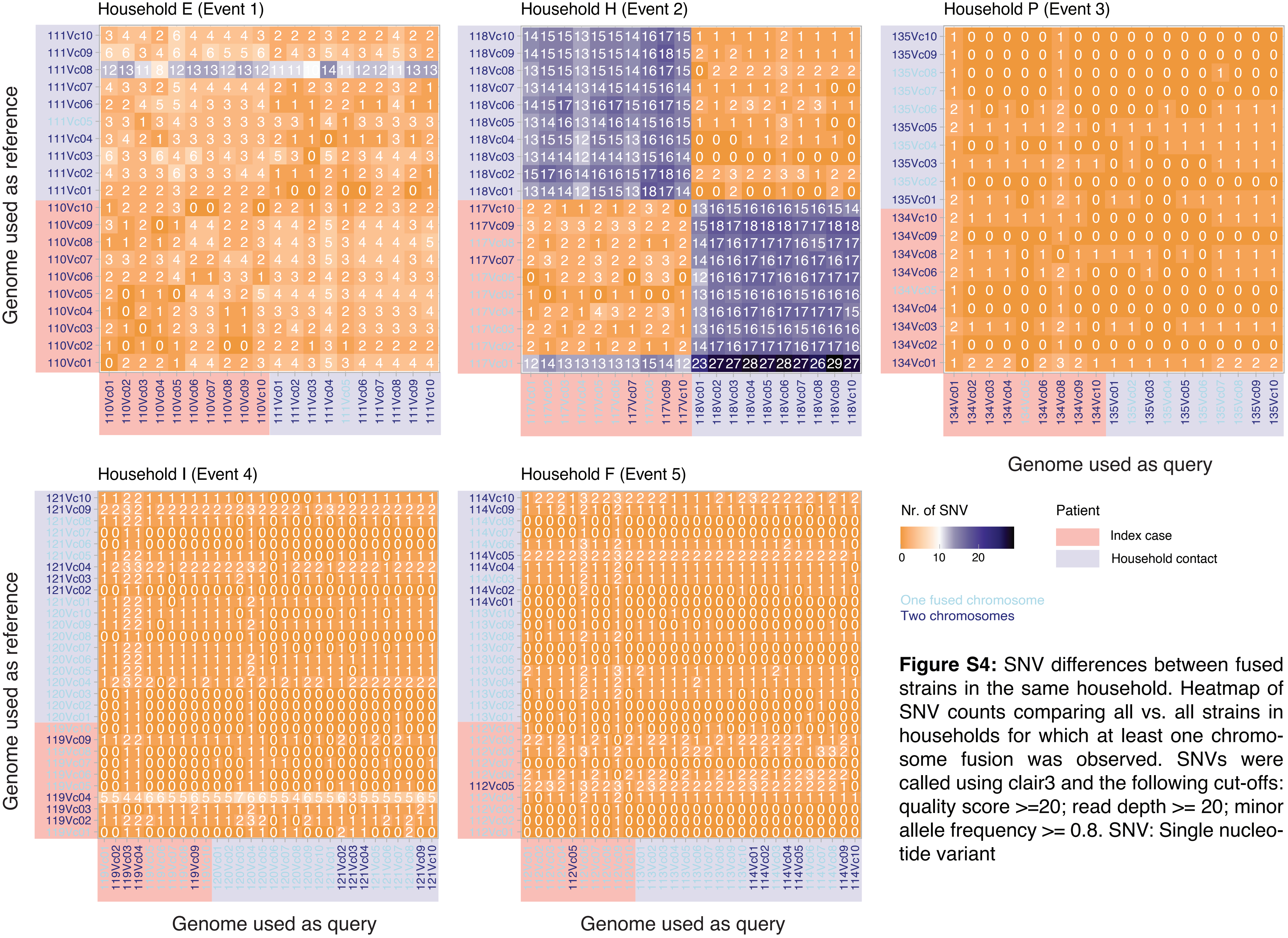

Figure S4: SNV differences between fused strains in the same household. Heatmap of SNV counts comparing all vs. all strains in households for which at least one chromosome fusion was observed. SNVs were called using clair3 and the following cut-offs: quality score >=20; read depth >= 20; minor allele frequency >= 0.8. SNV: Single nucleotide variant

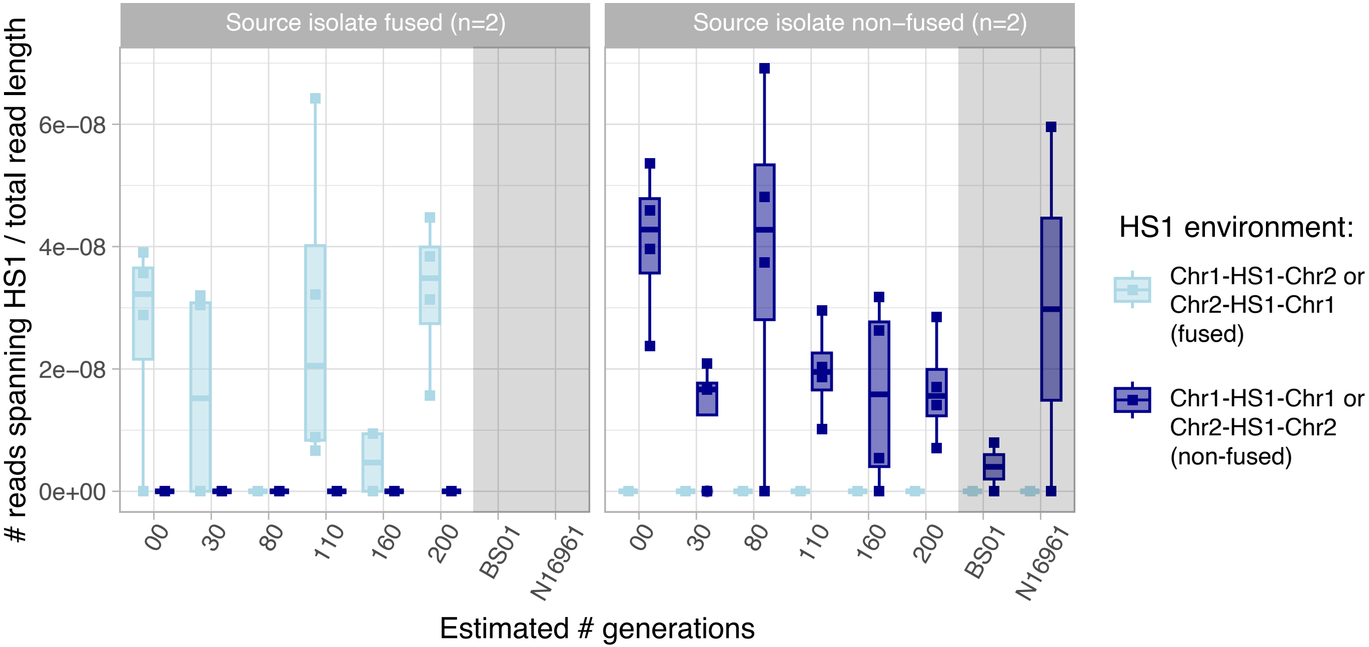

Figure S5: No evidence for fusion or fission after 200 generations of growth in the lab. Reads mapping against HS1 anchored in sequences of both chromosomes (fused state) or anchored in sequences of the same chromosome (non-fused state). Two isolates with a fused chromosome (left) and with non-fused chromosomes (right) served as source isolates. We included two well characterised non-fused isolates as references (highlighted in grey). Boxplots indicate the median and interquartile ranges with the whiskers representing the lower and upper interquartile range*1.5. Individual data points are represented as squares. Source data are provided as a Source Data file.

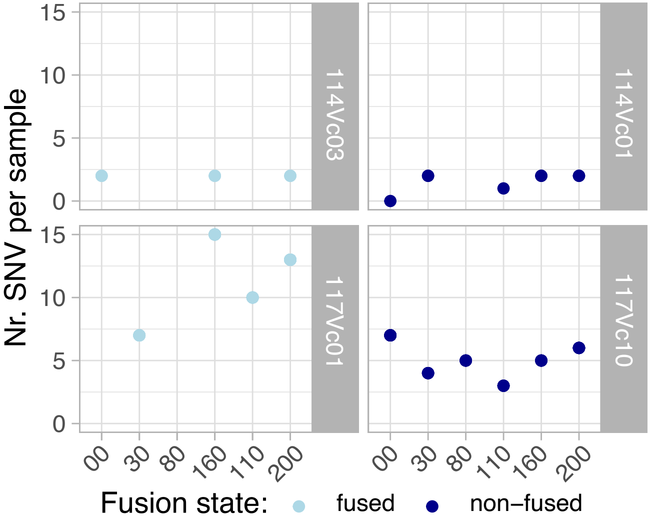

Figure S6: Few SNVs identified after 200 generations of growth in the lab. Single colonies were sequenced at different time points and SNVs to the source strain were called with clair3 (min. read depth = 10; min. allele frequency = 0.8; min. quality > 20). SNV= Sugle Nucleotide Variant. Source data are provided as a Source Data file.

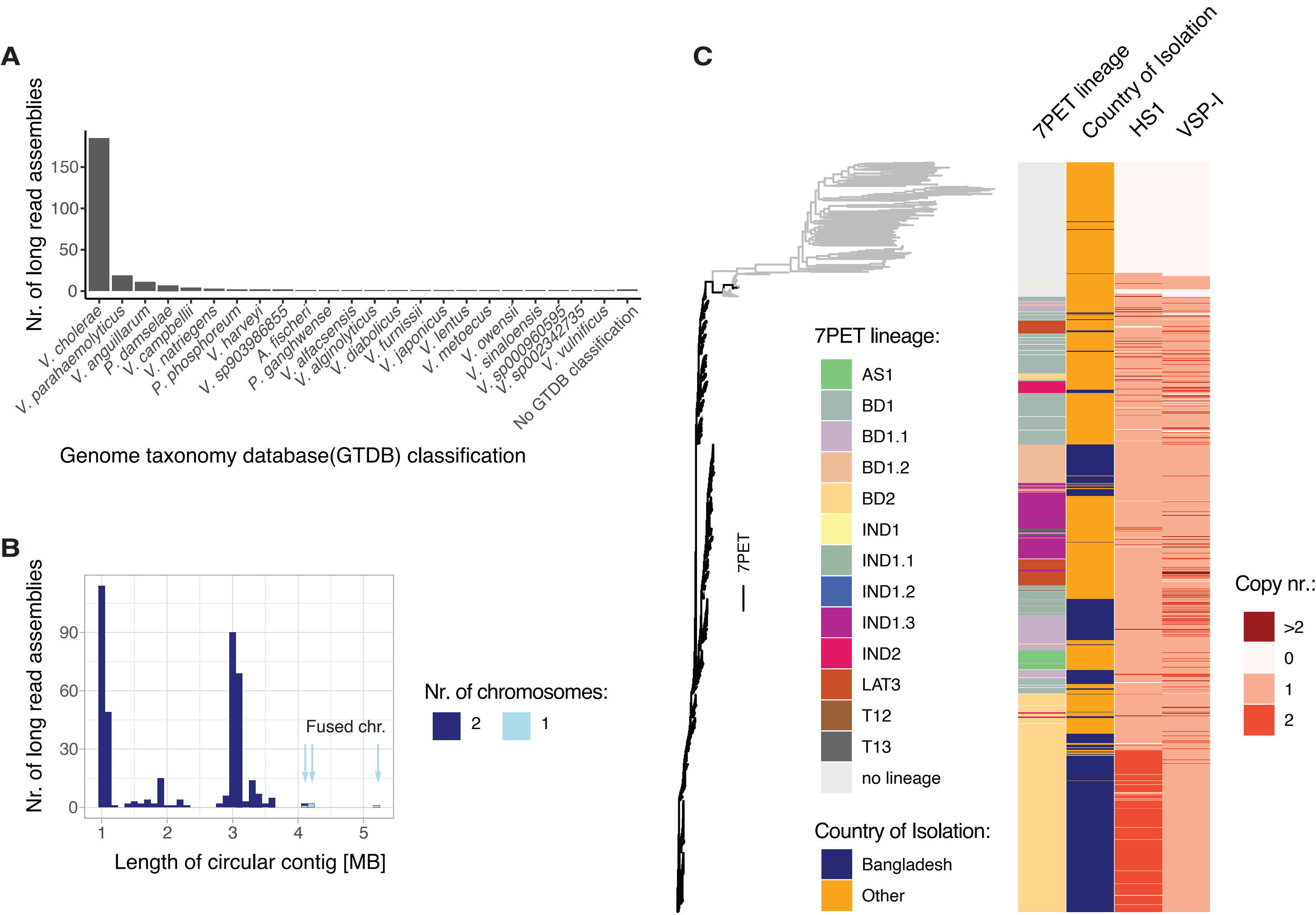

Figure S7: Chromosome fusion is rarely detected in other Vibrio genome sequences. A: *Vibrionales spp*. for which publicly available long-read assemblies were analysed **B:** Contig (chromosome) lengths of fully circularised genomes. Light blue arrows indicate fused chromosomes **C:** Core genome phylogeny of 1,223 publicly available short-read sequenced *Vc* genomes with heatmap indicating which 7PET lineage was assigned and the country of isolation and copy number estimations for HS1 and VSP-I. Whereas HS1 appears predominantly duplicated in the BD2 sublineage, VSP-I is more frequently duplicated in other 7PET sublineages. Source data are provided as a Source Data file.

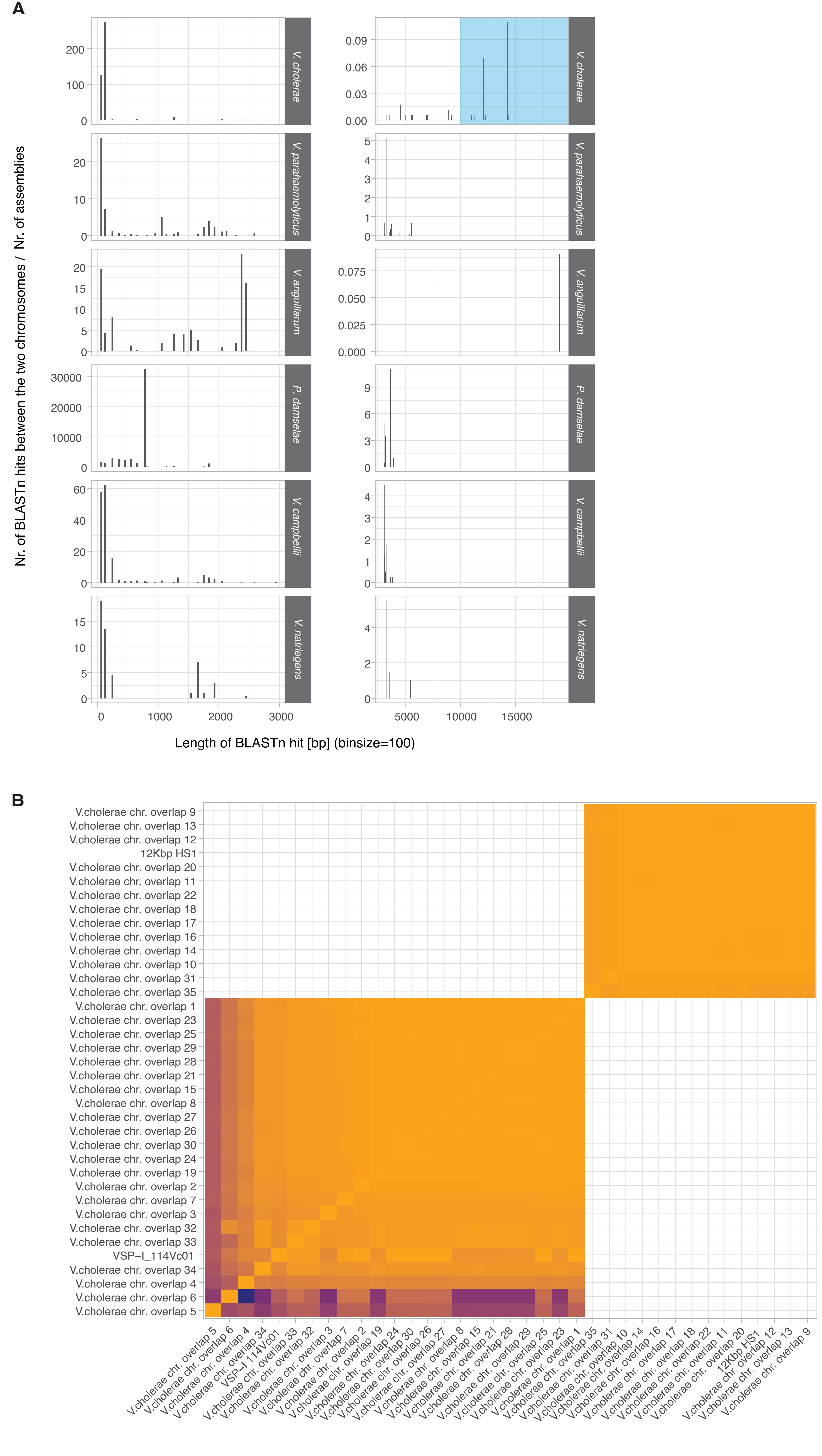

Figure S8: Chromosome comparison in bipartite genomes **A:** Number and length of BLAST hits with > 90% nucleotide identity between the chromosomes of each species. Left column: BLAST hits shorter than 3000 bp; right column: BLAST hits larger than 3000 bp. BLAST hits can be overlapping. Highlighted in blue are BLAST hits for *V. cholerae* longer than 10,000bp, which are included in B; **B:** Average Nucleotide Identities of the *V. cholerae* blast hits longer than 10,000bp and HS1 and VSP-I as references. bp: baise pairs; Source data are provided as a Source Data file.
