# Supplementary material for: Prevalent chromosome fusion in Vibrio cholerae O1
Source: Nat Commun. 2025 Jul 1;16:5830. doi: 10.1038/s41467-025-60699-0 (PMC12219848; doi:10.1038/s41467-025-60699-0)
Supplement: Supplementary file 2 — Description of Additional Supplementary Files [file 41467_2025_60699_MOESM2_ESM.pdf]

Description of Additional Supplementary Files:

Supplementary Data 1: Samples sequenced for this study and quality control measures of the sequences. Columns: "Median_read_length" = the median read length; "N50_reads": N50 of the reads (i.e. more than half of the data is in reads which have at least this length; "Median_read_quality_nanostat": Median read quality score as evaluated by Nanostat; "Mean_read_quality_nanostat": Mean read quality score as evaluated by Nanostat; "Read_depth": The evarage read depth of the assembly; "Contig_count": The number of assembled contigs; "Total_length_assembly": The total length of the assembly; "N50_assembly": The N50 of the contigs (half of the data is in contigs of at least this length; "GC_percent": % of GC in the assembly; "chr": The number of chromosomes assembled. "Individual": Participant identifyer; "Household" (household identifier); "Type_participant" (index case or household contact); "Symp" (whether the participant experienced symptoms or not); "Biosample" NCBI Biosample; "SRA accession": SRA accessionnumber of the raw reads.

Supplementary Data 2: Stability of fused and unfused chromosomes over 200 generations

Supplementary Data 3:

A: Publicly available long read Vibrionales spp. Sequences analysed for this study. Columns: "Sample": ENA accession number; "Median_read_length" = the median read length; "N50_reads": N50 of the reads (i.e. more than half of the data is in reads which have at least this length; "Median_read_quality_nanostat": Median read quality score as evaluated by Nanostat; "Read_depth": The evarage read depth of the assembly; "Contig_count": The number of assembled contigs; "Total_length_assembly": The total length of the assembly; "GC_percent": % of GC in the assembly; "species_classification": Species identified by GTDB-tk; "fraction_genus_bracken": the fraction of the assembly which was identified as belonging to the most abundant genus by kraken2 / bracken; "quality_control": Whether the genome passed the quality control and was included in further analysis.

B: Publicly available short read Vibrio cholerae sequences analysed for this study. Columns: "Sample": ENA accession number; "Read_quality": mean read quality; "Read_depth": The avarage read depth of the assembly; "Contig_count": The number of assembled contigs; "Total_length": The total length of the assembly; "GC_percent": % of GC in the assembly; "MetaPhlAn2_species": Species identified by Methaphlan2; "Year": Year of isolation; "Country": Country of isolation; "Dataset": In which dataset these genomes were included
