# Supplementary material for: Prevalent chromosome fusion in Vibrio cholerae O1
Source: Nat Commun. 2025 Jul 1;16:5830. doi: 10.1038/s41467-025-60699-0 (PMC12219848; doi:10.1038/s41467-025-60699-0)
Supplement: Supplementary file 6 — Reporting Summary [file 41467_2025_60699_MOESM6_ESM.pdf]

nature portfolio

Corresponding author(s): Aline Cuénod

Last updated by author(s): Apr 1, 2025

Reporting Summary

Nature Portfolio wishes to improve the reproducibility of the work that we publish. This form provides structure for consistency and transparency in reporting. For further information on Nature Portfolio policies, see our Editorial Policies and the Editorial Policy Checklist.

Statistics

For all statistical analyses, confirm that the following items are present in the figure legend, table legend, main text, or Methods section.

n/a	Confirmed	
☐	☒	The exact sample size (*n*) for each experimental group/condition, given as a discrete number and unit of measurement
☒	☐	A statement on whether measurements were taken from distinct samples or whether the same sample was measured repeatedly
☐	☒	The statistical test(s) used AND whether they are one- or two-sided<br>*Only common tests should be described solely by name; describe more complex techniques in the Methods section.*
☒	☐	A description of all covariates tested
☒	☐	A description of any assumptions or corrections, such as tests of normality and adjustment for multiple comparisons
☒	☐	A full description of the statistical parameters including central tendency (e.g. means) or other basic estimates (e.g. regression coefficient) AND variation (e.g. standard deviation) or associated estimates of uncertainty (e.g. confidence intervals)
☒	☐	For null hypothesis testing, the test statistic (e.g. *F*, *t*, *r*) with confidence intervals, effect sizes, degrees of freedom and *P* value noted<br>*Give P values as exact values whenever suitable.*
☒	☐	For Bayesian analysis, information on the choice of priors and Markov chain Monte Carlo settings
☒	☐	For hierarchical and complex designs, identification of the appropriate level for tests and full reporting of outcomes
☒	☐	Estimates of effect sizes (e.g. Cohen's *d*, Pearson's *r*), indicating how they were calculated

Our web collection on statistics for biologists contains articles on many of the points above.

Software and code

Policy information about availability of computer code

Data collection	The selection of publicly available genomes analysed in this study is described in detail in the Methods section 'Selection of publicly available sequences'. Sequences were downloaded using 'wget' (1.21.4).
Data analysis	Data analysis described in detail in the Methods section of this manuscript. Software code used to analyse the data presented in this study is available on GitHUb (https://github.com/acuenod111/Single_chromosome_Vc) and the files to reproduce the analysis and figures can be accessed via the OpenScienceFoundation (https://osf.io/xyfvg/).

For manuscripts utilizing custom algorithms or software that are central to the research but not yet described in published literature, software must be made available to editors and reviewers. We strongly encourage code deposition in a community repository (e.g. GitHub). See the Nature Portfolio guidelines for submitting code & software for further information.

Data

Policy information about availability of data

All manuscripts must include a data availability statement. This statement should provide the following information, where applicable:

- Accession codes, unique identifiers, or web links for publicly available datasets
- A description of any restrictions on data availability
- For clinical datasets or third party data, please ensure that the statement adheres to our policy

Data availability

Sequence data generated for this study (raw reads and assemblies) have been deposited on NCBI under the accession code PRJNA1121190. All sequence analysis data generated, which are required to reproduce the figures and analysis presented in this study are provided in the Source Data file and via the OpenScienceFoundation (https://osf.io/xyfvg/).

Code availability
Software code used to analyse the data presented in this study is available on GitHUb (https://github.com/acuenod111/Single_chromosome_Vc) and the files to reproduce the analysis and figures can be accessed via the OpenScienceFoundation (https://osf.io/xyfvg/).

Research involving human participants, their data, or biological material

Policy information about studies with human participants or human data. See also policy information about sex, gender (identity/presentation), and sexual orientation and race, ethnicity and racism.

Reporting on sex and gender	NA
Reporting on race, ethnicity, or other socially relevant groupings	NA
Population characteristics	NA
Recruitment	Stool and rectal swab were sampled from patients with cholera admitted to the International Centre for Diarrhoeal Disease Research, Bangladesh (icddr,b) Dhaka Hospital and from their household contacts, as described in prior studies. Patients presenting to the hospital with severe acute diarrhoea and a stool culture positive for V. cholerae O1 were considered index patients. Persons who shared the same cooking pot with an index patient for 3 or more days are considered household contacts and were enrolled within 6 hours of the presentation of the index patient to the hospital. Rectal swabs were collected daily from household contacts during a 10-day period after presentation of the index case. Household contacts underwent daily clinical assessment of symptoms. Household contacts were defined as infected if any rectal swab culture was positive for V. cholerae O1. V. cholerae serotypes were determined using slide agglutination testing with polyvalent and specific antisera as in prior studies.<br>We excluded patients below 2 years of age and above 60 years old or with major comorbid conditions. Rectal swabs and stool from the day of enrollment and follow-up time points were collected and placed immediately on ice after collection and stored at -80°C until DNA extraction.
Ethics oversight	The Ethical and Research Review committees of the icddr,b (approval number PR-11041) and the Institutional Review Boards of Massachusetts General Hospital, the University of Washington and McGill University (A07-M43-21B (21-07-026)) approved the study. All adult subjects in the study provided written informed consent and the parents/guardians of children provided written informed consent.

Note that full information on the approval of the study protocol must also be provided in the manuscript.

Field-specific reporting

Please select the one below that is the best fit for your research. If you are not sure, read the appropriate sections before making your selection.

- [x] Life sciences
- [ ] Behavioural & social sciences
- [ ] Ecological, evolutionary & environmental sciences

For a reference copy of the document with all sections, see nature.com/documents/nr-reporting-summary-flat.pdf

Life sciences study design

All studies must disclose on these points even when the disclosure is negative.

Sample size	We sequenced 467 Vibrio cholerae isolates collected from 47 patients living in 21 households in Dhaka, Bangladesh.<br>Samples sizes of V. cholerae strains tested in quantitative assays such as biofilm production, growth, and gene expression were determined based on the number of fused and non-fused chromosome pairs detected in our study. Replicates of these were determined based on the typical minimal variation between replicates we have in these assays. These are well-described and reproducible assays, and thus, we used replicates of 3 or more.
Data exclusions	No sequenced V. cholerae genome was excluded from the analysis.
Replication	For the wet lab experiments we clearly indicate the number of replicates for all experiments. Replication experiments performed confirmed findings and were reproducible.
Randomization	NA
Blinding	Blinding was not relevant to the study because quantitative, non-subjective outcomes were measured.

Reporting for specific materials, systems and methods

We require information from authors about some types of materials, experimental systems and methods used in many studies. Here, indicate whether each material, system or method listed is relevant to your study. If you are not sure if a list item applies to your research, read the appropriate section before selecting a response.

Materials & experimental systems

n/a	Involved in the study
☒	☐ Antibodies
☒	☐ Eukaryotic cell lines
☒	☐ Palaeontology and archaeology
☐	☒ Animals and other organisms
☒	☐ Clinical data
☒	☐ Dual use research of concern
☒	☐ Plants

Methods

n/a	Involved in the study
☒	☐ ChIP-seq
☒	☐ Flow cytometry
☒	☐ MRI-based neuroimaging

Animals and other research organisms

Policy information about studies involving animals; ARRIVE guidelines recommended for reporting animal research, and Sex and Gender in Research

Laboratory animals	NA
Wild animals	NA
Reporting on sex	NA
Field-collected samples	Bacterial (Vibrio cholerae) isolates sampled from patients living in Dhaka, Bangladesh.
Ethics oversight	The Ethical and Research Review committees of the icddr,b (approval number PR-11041) and the Institutional Review Boards of Massachusetts General Hospital, the University of Washington and McGill University (A07-M43-21B (21-07-026)) approved the study. All adult subjects in the study provided written informed consent and the parents/guardians of children provided written informed consent.

Note that full information on the approval of the study protocol must also be provided in the manuscript.

Plants

Seed stocks	NA
Novel plant genotypes	NA
Authentication	NA
