# Supplementary figures and images for: Prevalent chromosome fusion in Vibrio cholerae O1
Source: Nat Commun. 2025 Jul 1;16:5830. doi: 10.1038/s41467-025-60699-0 (PMC12219848; doi:10.1038/s41467-025-60699-0)

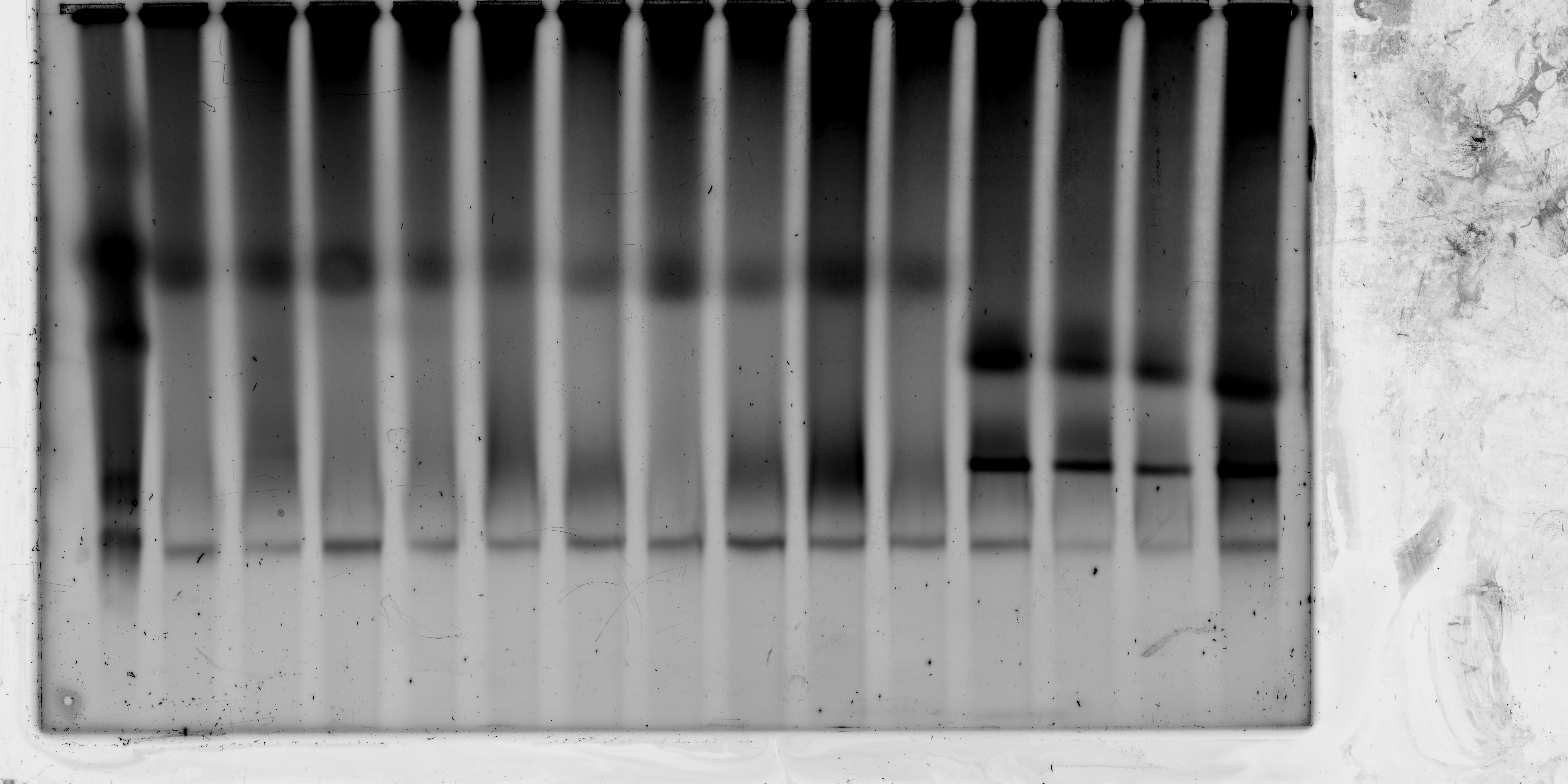

Supplement: Supplementary file 8 — Source Data [file 41467_2025_60699_MOESM8_ESM.zip › source_data/Figure_1/1b/rhyppa_040824_04-[Cy2].gel]
